# Molecular and Biological Characterization of Ralstonia Phage RsoM1USA, a New Species of *P2virus*, Isolated in the United States

**DOI:** 10.3389/fmicb.2019.00267

**Published:** 2019-02-19

**Authors:** Hardian Susilo Addy, Abdelmonim Ali Ahmad, Qi Huang

**Affiliations:** ^1^Floral and Nursery Plants Research Unit, United States National Arboretum, United States Department of Agriculture–Agricultural Research Service, Beltsville, MD, United States; ^2^Department of Plant Protection, Faculty of Agriculture, University of Jember, Jember, Indonesia; ^3^Department of Plant Pathology, Faculty of Agriculture, Minia University, El-minia, Egypt

**Keywords:** *Ralstonia solanacearum*, *Ralstonia* phage, isolation, characterization, *P2virus*, *Myoviridae*

## Abstract

The first *Ralstonia*-infecting bacteriophage from soil of the United States, designated RsoM1USA, was isolated from a tomato field in Florida. Electron microscopy revealed that phage RsoM1USA is member of the genus *P2virus* in the family *Myoviridae* with an icosahedral head of about 66 nm in diameter, a contractile tail of about 152 nm in length, and a long “neck.” Phage RsoM1USA infected 12 of the 30 tested *R. solanacearum* species complex strains collected worldwide in each of the three *Ralstonia* species: *R. solanacearum*, *R. pseudosolanacearum*, and *R. syzygii*. The phage completed its infection cycle 180 min post infection with a burst size of about 56 particles per cell. Phage RsoM1USA has a genome of 39,309 nucleotides containing 58 open reading frames (ORFs) and is closely related to Ralstonia phage RSA1 of the species *Ralstonia virus RSA1*. The genomic organization of phage RsoM1USA is also similar to that of phage RSA1, but their integrases share no sequence homology. In addition, we determined that the integration of phage RsoM1USA into its susceptible *R. solanacearum* strain K60 is mediated by the 3′ 45-base portion of the threonine tRNA (TGT), not arginine tRNA (CCG) as reported for phage RSA1, confirming that the two phages use different mechanism for integration. Our proteomic analysis of the purified virions supported the annotation of the main structural proteins. Infection of a susceptible *R. solanacearum* strain RUN302 by phage RsoM1USA resulted in significantly reduced growth of the infected bacterium *in vitro*, but not virulence in tomato plants, as compared to its uninfected RUN302 strain. Due to its differences from phage RSA1, phage RsoM1USA should be considered the type member of a new species with a proposed species name of *Ralstonia virus RsoM1USA.*

## Introduction

Bacterial wilt caused by *Ralstonia solanacearum* is one of the most destructive plant diseases in the world. The bacterium is known to infect more than 50 families of plants in tropical, subtropical, and warm temperate regions of the world (Hayward, 1991). *R. solanacearum* is considered a species complex because it contains strains with great genetic variability. It is traditionally classified into five races based on host range and five biovars based on biochemical properties. Using molecular methods, *R. solanacearum* species complex (referred to as “*R. solanacearum*” for historic reason in this study) has been divided into four phylotypes and 53 sequevars (Fegan and Prior, 2005), and most recently into three *Ralstonia* species: *R. solanacearum* (referred to as “the current *R. solanacearum*” in this study), *R. pseudosolanacearum*, and *R. syzygii* (Safni et al., 2014). *R. solanacearum* normally enters its plant hosts via openings in roots and colonizes the xylem vessels causing disturbance of water transport and resulting in the appearance of wilt symptoms when its cell densities are over 10^8^ CFU/cm stem (Huang and Allen, 2000). *R. solanacearum* is very difficult to control because of its wide host range, large genetic diversity and its capability for long-term survival in soil and water. It is, therefore, desirable to explore alternative control strategies such as the use of bacteriophages to combat *R. solanacearum*. Bacteriophages that have been tested so far for control of *R. solanacearum* have recently been reviewed by Alvarez and Biosca (2017).

Currently, a wide variety of bacteriophages that specifically infect *R. solanacearum* have been characterized and belong to the families *Inoviridae*, *Myoviridae*, *Podoviridae*, or *Siphoviridae*. Interestingly, all *Ralstonia*-infecting bacteriophages reported so far were isolated from soil collected from Asian countries including Japan (Yamada et al., 2007; Van et al., 2014; Thi et al., 2015; Bhunchoth et al., 2016; Askora et al., 2017), Thailand (Bhunchoth et al., 2015, 2016; Mihara et al., 2016), Korea (Murugaiyan et al., 2010; Park, 2018), China (Su et al., 2017; Liao, 2018), and Indonesia (Addy et al., 2018), except for one from Egypt (Ahmad et al., 2018) and another not from soil but from the culture supernatant of *R. solanacearum* strain UW551 (Ahmad et al., 2017b). Six *Ralstonia* phages in the family of *Myoviridae* have been characterized including RSA1 (Yamada et al., 2007; Fujiwara et al., 2008), RSL1 (Yamada et al., 2007, 2010), RSF1 (Bhunchoth et al., 2016), and RSY1 (Askora et al., 2017) isolated from soil in Japan, as well as RP15 (Mihara et al., 2016) and RSL2 (Bhunchoth et al., 2016) in Thailand. Among the six myoviruses, RSL1, RSL2, and RSF1 are jumbo phages with genome sizes over 220-kb and RP15 has a genome of 168-kb, while RSA1 and RSY1 are relatively small-genome phages of 39- and 40-kb in size, respectively. Although phages RSA1 and RSY1 are similar in genome size and genomic organization, they differ in the orientation of the integrase gene. In addition, RSY1 uses part of serine tRNA (GGA) gene, while RSA1 uses a portion of arginine tRNA (CCG) gene as the *att* region, resulting in the grouping of phages into RSA1-type and RSY1-type of the P2-like phages of *R. solanacearum* (Askora et al., 2017). Both the jumbo phage RSL1 and the small-genome phage RSA1 were characterized biologically for their biocontrol potential for *R. solanacearum*. They both displayed lytic activities against almost all tested *R. solanacearum* strains (belonging to the current *R. pseudosolanacearum* strains) isolated from Japan (Yamada et al., 2007) and reduced host bacterial cell density (Fujiwara et al., 2011). Populations of resistant *R. solanacearum* cells, however, were observed 30 h after phage RSA1 was added to the bacterial culture (Fujiwara et al., 2011), rendering RSA1 not suitable as a biocontrol for *R. solanacearum*. Subsequent greenhouse experiments with phage RSL1 demonstrated that this phage has a good potential for control of bacterial wilt caused by *R. solanacearum* (Fujiwara et al., 2011), although its potential against non-Japanese *R. solanacearum* species complex strains is unknown. These studies suggest that bacteriophages, even in the same virus family and that display similar *in vitro* lytic activities against large numbers of tested *R. solanacearum* strains, have different potential *in planta* as biocontrol agents, and detailed biological characterization of phages are needed before their biocontrol potential can be assessed.

In this study, we purified and characterized the first *Ralstonia*-infecting bacteriophage from soil samples collected from the United States, isolated from a tomato field in Florida. We also compared the phage to other bacteriophages, determined its integration mechanism, and evaluated its effect *in vitro* and *in planta* on its susceptible *R. solanacearum* host strain under controlled environment conditions.

## Materials and Methods

### *R. solanacearum* Strains

Thirty *R. solanacearum* species complex strains used in this study are listed in Table 1. They were grown, maintained and their inocula prepared as described before (Stulberg et al., 2015).

**Table 1:** Susceptibility of *R. solanacearum* species complex strains to phage RsoM1USA.

*R. solanacearum* species complex strain		Biovar, phylotype-sequevar	Origin	Susceptibility to phage RsoM1USA based on	
				
				Spot test^a^	EOP^b^
*R. solanacearum*	RUN302	1, IIB-4	Brazil	S	10^0^
	K60	1, IIA-7	United States	S	5 × 10^-1^
	P550	1, IIA-7	United States	S	6 × 10^-2^
	Rs5	1, IIA-7	United States	R	
	RUN074	1, IIB-3	Philippines	S	8 × 10^-3^
	AW1	1, IIA-7	United States	R	
	RUN036	1, IIA-36	Martinique	R	
	RUN651	1, IIB-4	France	R	
	4153	2, II	United Kingdom	S	3 × 10^-2^
	Pss1475	2, II	Taiwan	S	8.3 × 10^-2^
	RUN035	2, IIB-1	Netherlands	S	4.3 × 10^-2^
	RUN133	2, II-29	Cameroon	R	
	UW224	2, IIB-1	Kenya	R	
	UW257	2, IIB-1	Costa Rica	R	
	UW276	2, II	Mexico	R	
	UW344	2, IIB-1	Brazil	R	
	UW425	2, II	Australia	R	
	UW550	2, IIB-1	Netherlands	S	2 × 10^-2^
	UW551	2, IIB-1	Kenya	R	
	UW552	2, IIB-1	Guatemala	S	1 × 10^-1^
	UW349	2T, IIB-27	Brazil	S	4 × 10^-1^
*R. pseudosolanacearum*	DT3	3, I	Indonesia	R	
	GMI1000	3, I-18	French Guiana	R	
	HB512	3, I	China	R	
	Pss4	3, I-15	Taiwan	S	8 × 10^-2^
	Pss530	3, I	Taiwan	S	8 × 10^-2^
	Rs121	3, I	United States	R	
	UW152	3	Australia	R	
	Ps191	4, I	Taiwan	R	
*R. syzygii*	RUN083	2T, IV-10	Indonesia	R	


*^*a*^ Susceptibility of *R. solanacearum* strains to phage RsoM1USA is shown as resistant (R) when no plaques were observed or susceptible (S) when clear plaques were observed. Phage spot test was performed using purified phage first, and the potentially susceptible strains were tested further by serial dilution plaque assay to confirm their susceptibility. ^*b*^Efficiency of plating (EOP) values were calculated by dividing the phage titer on a given Ralstonia strain by the titer on strain RUN302, which showed the highest titer compared to other tested strains.*

### Bacteriophage Isolation, Purification, and Characterization

Soil samples from a tomato field infested by *R. solanacearum* in Florida, United States were used for phage isolation. Ten grams of soil were mixed with water for a total volume of 30 ml in a sterile 50-ml centrifuge tube with gentle shaking overnight at room temperature to release bacteriophages. The tube was then centrifuged at 8,000 × *g* for 20 min at room temperature, and the supernatant was filtered through a 0.45-μm membrane. A 3 μl aliquot of the filtrate was spotted on a double-layered casamino acid peptone glucose (CPG) plate (Ahmad et al., 2017b) except that *R. solanacearum* strain RUN302 was used as a bacterial host. The plate was incubated at 28°C for 24 h for the appearance of clear zones caused by phages. A single clear zone was removed, placed into 1 ml of SM buffer (100 mM NaCl, 10 mM MgSO_4_, 50 mM Tris-HCl at pH 7.5, and 0.01% gelatin) (Sambrook and Russell, 2001), and vortexed vigorously prior to filtering through a 0.45-μm membrane. The filtrate was then serially diluted with the SM buffer. One hundred microliters of the diluted filtrate was added to 400 μl of 2 × 10^8^ cells of *R. solanacearum* strain RUN302, incubated for 10–20 min at 28°C for phage absorption, and mixed with 3.5 ml of CPG containing 0.45% agar before layering the mixture on top of a CPG plate containing 1.5% of agar for the plaque assay to obtain single plaques. A single plaque was then picked, mixed with SM buffer, vortexed, filtered, serially diluted, and subjected to the plaque assay described above. The process was repeated two more times to complete the triple phage purification process to obtain a pure phage isolate designated RsoM1USA. To make a pure phage stock, a single plaque of phage RsoM1USA was removed and added to a 5 ml liquid CPG culture of 2 × 10^8^ cells of *R. solanacearum* strain RUN302. It was grown overnight at 28°C with shaking, followed by centrifugation at 8,000 × *g* for 20 min. The supernatant was filtered through a 0.45 μm membrane, and the filtrate stored in the dark at 4°C. The titer and the optimum multiplicity of infection (MOI) of the phage stock were determined using the plaque assay described above.

The phage particles were propagated, purified, and stored as described (Ahmad et al., 2017b), except that *R. solanacearum* strain RUN302 was used as the host strain. Briefly, the phage lysate was first cleared by low speed centrifugation (8,000 × *g* for 15 min), passed through a 0.45 μm membrane filter, and then layered on top of a 30% (wt/vol) sucrose cushion before ultracentrifugation at 50,000 × *g* for 120 min at 10°C. To obtain a phage titer of about 10^10^ PFU/ml, the purification process may need to be repeated two or three times using the same volume of lysate each time and by ultracentrifugation through a 30% sucrose cushion in the same tube where the pellet from previous ultracentrifugation was collected. The pellet was then resuspended in either TE or SM buffer.

To characterize the morphology of the phage, purified phage particles were used for negative staining with sodium phosphotungstate (Dykstra, 1993) before observation under a Hitachi HT7700 transmission electron microscope. Phage morphometrics were estimated from more than 10 phage particles using the open source imaging processing program ImageJ 1.50i (National Institutes of Health, United States).

### Phage Host Range Test

To determine the host specificity of phage RsoM1USA, the purified phage was subjected to spot testing using 30 *R. solanacearum* species complex strains as hosts (Table 1). In this test, three microliters of the phage suspension (10^8^ PFU/ml) was spotted on top of the double-layered CPG plate (Ahmad et al., 2017b). The top layer was prepared with 350 μl of each *R. solanacearum* strain (OD_600_ of 0.2) mixed with 4.5 ml of CPG. The plate was incubated overnight at 28°C. The formation of a lysis zone indicated that the bacterial strain was susceptible to the phage. Potentially susceptible strains were tested further by serial dilution plaque assay to determine if they were truly susceptible to the phage. In addition, the efficiency of plating (EOP) was determined based on Gill et al. (2011) by calculating the ratio of the phage plaque titer of *R. solanacearum* strain RUN302 to that of other tested bacterial strains.

### One-Step Growth Experiment

The phage infection cycle was characterized with a one-step growth experiment based on Ellis and Delbruck (1939) with modifications. Two hundred microliters of 24-h culture of *R. solanacearum* strain RUN302 was transferred into 9.8 ml of CPG and grown at 28°C with shaking until the culture reached the OD_600_ of 0.1 (10^8^ CFU/ml). Phage RsoM1USA was added at a MOI of 0.01 and allowed to adsorb for 15 min at 28°C. The mixture was then centrifuged at 6,000 × *g* for 5 min to remove any non-absorbed phage particles. The pellet was mixed with 10 ml of CPG, diluted 10,000-fold, and incubated at 28°C without shaking. An aliquot of 500 μl was taken every 30 min for 5 h, filtered through 0.45 μm membrane, diluted, and subjected to the plaque assay described above using RUN302 as a host to estimate phage titers. There were three replicates for each time point, and the experiment was repeated three times.

### Thermal Stability Test

To determine the effect of temperature on phage stability, a thermal stability test was conducted at 4°C, and from 10–90°C with 10° intervals. The maximum of 90°C was tested to find out the lethal temperature for the phage, not as a temperature for biocontrol application. Briefly, the purified phage in SM buffer was diluted to 1 × 10^8^ PFU/ml and then 1 ml of the diluted phage suspension was incubated at each of the designated temperatures for 1 h. After incubation, the phage suspension was serially diluted in SM buffer and subjected to plaque assay using *R. solanacearum* RUN302 as a host to estimate phage numbers. There were three replicates for each temperature and the experiment was repeated three times.

### Phage DNA Extraction, Sequencing, and Analysis

To determine the nature of phage RsoM1USA genome, approximately 1 μg of the phage genome was subjected to enzyme digestions with DNase I, RNase A, S1 nuclease, and exonuclease I as described (Ahmad et al., 2017b), as well as with restriction enzymes *Eco*RV and *Sma*I using standard molecular biology method (Sambrook and Russell, 2001).

Phage DNA was extracted from purified phage particles (described above) using either a phenol–chloroform method (Sambrook and Russell, 2001) or the Phage DNA Isolation kit (Norgen Biotek Corp., Canada). The phage DNA was sequenced, and the genome sequence assembled commercially by SeqMatic (Fremont, CA, United States). Potential open reading frames (ORFs) larger than 50 amino acids (aa) were identified using the softwares PHASTER (Arndt et al., 2016) and DNASTAR (DNASTAR Inc., United States). The number of aa and the predicted molecular weight for the product of each ORF were calculated using the software ApE version 2.0.51 (University of Utah, United States). To assign possible functions of the ORFs, database searches were performed using BLASTp (Altschul et al., 1990) against NCBI and the Conserved Domain Databases, as well as against motif databases including TIGRFam, Pfam, SMART, PRK, COG and InterPro. The tRNAscan-SE 2.0^1^ was used to search for tRNA genes (Lowe and Eddy, 1997).

Phylogenetic relationships between phage RsoM1USA and 13 phages representing each of the 13-current species in the genus *P2virus* under the subfamily *Peduovirinae* of the family *Myoviridae* were estimated. This was done using the method of Harrison and Langdale (2006) by multiple sequence alignment using Clustal Omega^2^ (Sievers et al., 2011) to generate nexus-formatted files, followed by execution of the files using downloadable PAUP (Phylogenetic Analysis Using PAUP^3^) version 4.0a (Swofford, 2003). Phylogenetic analysis based on integrase was performed for 13 phages including RsoM1USA, since no annotation for an integrase has been found for *Burkholderia virus phiE122.* The accession and ORF numbers of the proteins used for the phylogenetic analysis are listed in Table 2.

**Table 2:** List of proteins and their ORFs or accession numbers of bacteriophages used for phylogenetic analysis in this study.

Bacteriophage	ORF # for phage RsoM1USA and accession # for other phages			
	
	Phage capsid protein	Terminase ATPase subunit protein	Phage portal protein	Integrase
Ralstonia phage RsoM1USA	MG747435	MG747435	MG747435	MG747435
	(ORF9)	(ORF7)	(ORF6)	(ORF 56)
*Ralstonia virus RSA1*	YP_001165257.1	YP_001165255.1	YP_001165254.1	YP_001165299.1
*Mannheimia virus PHL101*	YP_655472.1	YP_655470.1	YP_655469.1	YP_655517.1
*Burkholderia virus phi52237*	YP_293748.1	YP_293751.1	YP_293752.1	YP_293708.1
*Burkholderia virus phiE122*	ABO60795.1	ABO60770.1	ABO60792.1	–^a^
*Burkholderia virus phiE202*	YP_001111033.1	YP_001111035.1	YP_001111036.1	YP_001111041.1
*Pseudomonas virus phiCTX*	NP_490602.1	NP_490600.1	NP_490599.1	NP_490644.1
*Salmonella virus PsP3*	NP_958060.1	NP_958058.1	NP_958056.1	NP_958084.1
*Salmonella virus SopEphi*	AAQ65016.1	AAQ65014.1	AAQ65013.1	AAQ64997.1
*Salmonella virus Fels2*	YP_001718745.1	YP_001718747.1	YP_001718748.1	YP_001718763.1
*Escherichia virus P2*	NP_046760.1	NP_046758.1	NP_046757.1	NP_046786.1
*Escherichia virus 186*	NP_052253.1	NP_052251.1	NP_052249.1	NP_052278.1
*Escherichia virus Wphi*	AAN28222.1	AAN28220.1	AAN28219.1	AAN28248.1
*Yersinia virus L413C*	NP_839853.1	NP_839851.1	NP_839850.1	NP_839878.1


*^*a*^Annotation for an integrase was not found in the phage sequence.*

### Identification and Proteomic Analysis of RsoM1USA Virion Proteins

Fifty milliliters of purified phage filtrate were treated with 1 μl each of 10 mg/ml RNase A and DNase I for an hour at 37°C prior to adding chloroform at a final concentration of 10%, followed by centrifugation at 7,000 × *g* for 10 min at 4°C. The top phase containing phage particles was layered on a 20% (w/v) sucrose cushion and centrifuged by ultracentrifugation (Optima MAX-XP Ultracentrifuge, Beckman Coulter, United States) at 50,000 × *g* for 2 h at 4°C. The phage pellet was suspended in 500 μl of 10 mM Tris-HCl buffer pH 8. Aliquots from purified phage particles was sonicated, mixed with Norex^TM^ tris glycine SDS buffer (2x) (Thermo Fisher Scientific, United States), heated at 85°C for 2 min, and subjected to SDS-PAGE [14% (w/v) polyacrylamide] (Laemmli, 1970). Gels were stained with Coomassie Brilliant Blue R250 stain reagent (Thermo Scientific, United States). The most abundant bands were cut out and sent to Bioproximity, LLC. (Chantilly, VA, United States) for protein identification using liquid chromatography-tandem mass spectrometry (LC-MS/MS).

### Assay to Determine Phage Integration Regions

*R. solanacearum* strain K60 was used to determine where phage RsoM1USA integrated into the bacterial genome, since the complete genome sequence of K60 is publicly available (Hayes et al., 2017). In addition, the bacterial strain did not contain prophage sequence of RsoM1USA in its genome and is susceptible to the phage infection. To infect strain K60, an overnight culture of K60 was adjusted to OD_600_ of 0.1 in 4.5 ml of liquid CPG. Phage RsoM1USA was then added at a MOI of 0.1 and adsorbed to bacterial cells for 15 min at 28°C, before incubation at 28°C with shaking at 150 rpm for 24 h. The infected bacterial cells were harvested by centrifugation at 6,000 × *g* for 10 min at 4°C, and the pellet was suspended in sterile water and used for DNA extraction as described by Sambrook and Russell (2001). PCR primers designed and used for this study are listed in Table 3. The primers were designed based on the nucleotide sequences flanking the predicted *att*P site in phage RsoM1USA and *att*B site in *R. solanacearum* strain K60.

**Table 3:** List of primer pairs designed and used in this study.

Primer pair	Sequence (5′–3′)	Position in phage RsoM1USA or *R. solanacearum* strain K60^∗^ (italicized)	Size of PCR product (bp)	Target
tRNA-349F	CAGTCTGTGTACGACGTGGC	36,097–36,116	1,068	*att*P
tRNA-349R	CGGACAAAGCCCTCTTCGAC	37,145–37,164		
attB-K60-F	*AAACTGTCCGCTGTGGAGTC*	*2,473,579–2,473598*	452	*att*B
attB-K60-R	*CCCTTTGATGCGTTGGTACT*	*2,474,011–2,474,030*		
attB-K60-F	*AAACTGTCCGCTGTGGAGTC*	*2,473,579–2,473598*	720	*att*L
tRNA-349R	CGGACAAAGCCCTCTTCGAC	37,145–37,164	
tRNA-349F	CAGTCTGTGTACGACGTGGC	36,097–36,116	800	*att*R
attB-K60-R	*CCCTTTGATGCGTTGGTACT*	*2,474,011–2,474,030*		


*^∗^ Accession no: NCTK01000001.1.*

PCR was performed in a 20-μl volume containing 1x GoTaq Green Master Mix (Promega, United States), 10 pmol of each primer, and 25 nanograms of DNA. PCR conditions were 1 cycle of 1 min at 94°C, 30 cycles of 30 s at 94°C, 30 s at 60°C, and 1 min at 72°C with a final extension of 10 min at 72°C. The PCR products were purified from agarose gels with the QIAquick gel extraction kit (Qiagen, Inc.) and sequenced by Eurofins Scientific (United States).

### Assay for *in vitro* Growth of Phage-Treated *R. solanacearum* Strains

To determine the effect of phage infection on the *in vitro* growth of its susceptible *R. solanacearum* strain, phage RsoM1USA-treated and untreated *R. solanacearum* RUN302 strains were grown in CPG in 24-well plates (Corning^®^ Sigma, United States) at 28°C. Briefly, the concentration of the overnight culture of strain RUN302 was adjusted with CPG to approximately 10^8^ CFU/ml, and 1.5 ml of the bacterial suspension was added to each well of the 24-well plate. One hundred and fifty microliters of phage suspension were then added at a MOI of 0.001, 0.01, 0.1, 1.0, and 10, respectively, and the plate was incubated inside a multi-plate reader Epoch2 (BioTek, United States) with slow shaking. SM buffer was used as a phage control (MOI of 0). Bacterial growth indicated by absorbance at 600 nm was measured every 60 min and graphed every 5 h for 35 h. There were three replicates for each MOI treatment and the experiment was repeated three times.

### Virulence Assay in Tomato Plants

Tomato plants (*Lycopersicon esculentum* Mill. cv. ‘Bonnie Best’) were grown, transplanted and inoculated as described previously (Ahmad et al., 2017b), except that 40 ml of bacterial suspension containing a total of 4 × 10^9^ cells of *R. solanacearum* strain RUN302 was added as a soil drench into each pot. This was followed immediately by pouring 40 ml of phage RsoM1USA suspension (10^7^ PFU/ml) for a MOI of 0.1 into the pot, or water for a control (MOI of 0). Negative control plants were inoculated with 80 ml of water. Inoculated plants were rated daily using a disease index (DI) of 0 to 4 (Roberts et al., 1988). Disease severity was calculated each day using the formula: Disease severity = [Σ(*n* × DI)/4*N*] × 100, where *n* is the number of plants in each DI category and *N* is the total number of plants in each treatment. There were 12 plants for each treatment and the experiment was repeated three times.

### Statistical Analysis

Data for phage thermal stability and *in vitro* growth of *R. solanacearum* strains 35 h after phage RsoM1USA treatment at MOIs of 0.001, 0.01, 0.1, 1, and 10 were analyzed by one-way ANOVA using free web-based statistical software^4^. Tukey’s Honest Significant Difference test included in the software was used to compare means. The mean disease severity between the untreated and phage RsoM1USA-treated *R*. *solanacearum* RUN302 strains were analyzed for significant differences using the *t*-test in Microsoft Excel. The *t*-test was also performed for *in vitro* growth of *R. solanacearum* strain RUN302 between RsoM1USA-untreated (MOI of 0) and treated (MOI of 0.001, 0.01, 0.1, 1 or 10) strains 10, 15, and 35 h after phage treatment. Differences were considered statistically significant if *p* < 0.05.

### Nucleotide Sequence Accession Numbers

The complete genome sequence of phage RsoM1USA has been submitted to GenBank and given accession no. MG747435. The accession numbers for *R. solanacearum* strain sequences used in this study are: GMI1000, NC_003295; 23-10BR, JQOI01000031.1; Po82, NC_017574.1; and K60, NCTK01000001.1.

## Results

### Morphology, Host Specificity, and Growth Characteristics of Ralstonia Phage RsoM1USA

**FIGURE 1:**
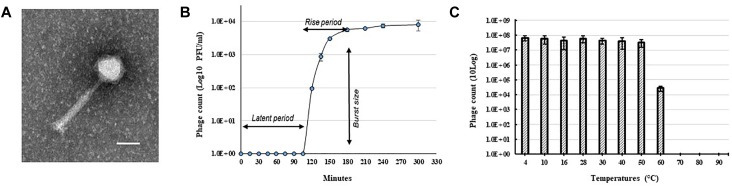
Morphology and growth characteristics of Ralstonia phage RsoM1USA. **(A)** Transmission electron micrograph of the purified phage virion. The scale represents 50 nm. **(B)** One-step growth curve of the phage with *R. solanacearum* strain RUN302 as the host. The phage was added at a MOI of 0.01 and allowed to adsorb for 15 min at 28°C. Phage titers were determined every 30 min using the plaque assay. The latent period is when no release of phage particles was observed. The rise period begins with the end of the latent period and ends when the increase in phage titers ceases. The burst size is the average number of phage particles released per infected cell. **(C)** Effect of temperature on the stability of phage RsoM1USA. 10^8^ PFU of phage RsoM1USA was incubated at each temperature point and the number of phage was estimated by plaque assay using *R. solanacearum* RUN302 as a host 1 h after incubation. Means are based on three separate experiments, each containing three replicates. Bars indicate standard errors.

A bacteriophage isolated from soil collected from a tomato field in Florida, United States produced clear and round plaques with a diameter of approximately 5 mm on double layered CPG plates using *R. solanacearum* strain RUN302 as a host. The purified phage had an icosahedral capsid with a size of 62.68 ± 2.17 nm × 66.17 ± 2.61 nm (*n* = 10) and a long contractile tail with a length of 151.96 ± 4.92 nm and a width of 19.63 ± 0.72 nm (*n* = 10) (Figure 1A). In addition, the phage has a relatively long “neck” (Figure 1A) compared to other myoviruses such as RP15 (Mihara et al., 2016) and the diameter of its sheath increased approximately 3 nm (*n* = 12) during contraction, resembling the members of *Peduovirinae*. The phage was designated Ralstonia phage RsoM1USA using our phage identifier naming system (Ahmad et al., 2018) based on the informal guidelines by Adriaenssens and Brister (2017) and the proposed scheme by Kropinski et al. (2009), since it is the first *R. solanacearum*-infecting bacteriophage belonging to the family *Myoviridae* that was isolated from the United States.

To determine the host specificity of phage RsoM1USA, 30 *R. solanacearum* strains originally isolated from different geographic locations of the world were tested for their susceptibility to the phage (Table 1). Phage RsoM1USA infected 12 of the 30 tested strains, including members of biovars 1, 2, 2T, and 3 belonging to each of the current *R. solanacearum* and *R. pseudosolanacearum* species (Table 1). The efficiency of phage plating was also determined for the 12 susceptible strains, and strain RUN302 was found to have the highest efficiency of phage infection (Table 1). The infection cycle of the phage was determined to be 180 min, including a 105 min of latent period, followed by a 75-min rise period with a burst size of 56 ± 6 particles per infected cell (Figure 1B). Phage RsoM1USA was stable from 4 to 50°C, since its titer at that temperature range remained similar at approximately 10^8^ PFU/ml (Figure 1C). Significant loss in the phage titer, however, was observed at 60°C, and no phage particles were detected after they were incubated at 70, 80, and 90°C for 1 h (Figure 1C).

### Genome Characterization of Ralstonia Phage RsoM1USA

**FIGURE 2:**
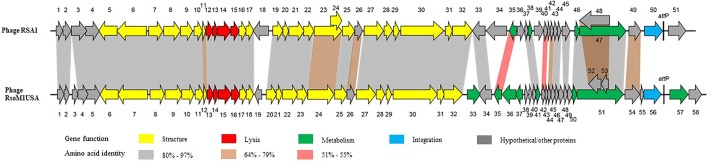
Comparison of genomic organization between phages RsoM1USA and RSA1. Different functional gene groups are represented and different levels of amino acid sequence identity between homologous ORFs are shaded with different colors. The location of the core attachment *att*P site is indicated in the two phages.

The genome of phage RsoM1USA1 was degraded by DNase I, but not by RNase A, Exonuclease I and S1 nuclease (data not shown). The genome was also digested into multiple bands when treated with restriction enzymes *Eco*RV and *Sma*I (data not shown). The complete nucleotide sequence of the Ralstonia phage RsoM1USA was determined and submitted to GenBank (accession no. MG747435). The phage genome consists of 39,309 bp with a G+C content of 65.33%. Fifty-eight potential ORFs were identified in phage RsoM1USA (Supplementary Table S1). Among them, 48 started with methionine (ATG), four with leucine (CTG for ORFs 1 and 52 and TTG for 18 and 36), and five with valine (GTG) for ORFs 5, 14, 21, 23, and 37). ORF35, however, started with GTC (valine), an unusual start codon described by Feeney et al. (2017). Forty-two ORFs ended with TGA, nine with TAG (ORFs 2, 18, 22, 23, 26, 29, 52, 57, and 58) and seven with TAA (ORFs 6, 28, 33, 36, 39, 45, and 5*5*). When the whole genome sequence of phage RsoM1USA was used as a query to search GenBank by BLASTn, the phage was found to be most closely related to *R. solanacearum* phage RSA1 (Yamada et al., 2007; Fujiwara et al., 2008), the member of the species *Ralstonia virus RSA1* in the genus *P2virus*, with 89% nucleotide identity and 79% coverage. Each of the 58 ORFs of phage RsoM1USA was used as a query to search GenBank by BLASTp, and their positions, and predicted functions are summarized in Supplementary Table S1. Each of the ORFs was also compared to ORFs of ϕRSA1 and their similarity levels are indicated in Figure 2.

### Gene Organization of Phage RsoM1USA and Comparison to Phage RSA1

The predicted ORFs of phage RsoM1USA can be categorized into the following four functional gene groups: metabolism (transcription and regulatory), structure (head and tail), lysis and integration, as well as hypothetical and other proteins.

#### (i) Metabolism

Eight ORFs were annotated to play a role in the phage’s metabolism, including ORF33 for a site-specific DNA-methyltransferase, ORF35 for a XRE family transcriptional regulator, ORF36 for a transposase, ORF37 for an insertion element repressor, ORF41 for phage transcription activator Org/Delta, a member of the zinc finger recombinase superfamily (pfam04606), ORF50 for a RNA binding protein, and ORF51 for a DNA primase. ORF57 was annotated to encode a ParA family protein, which were identical to its counterpart in *R. solanacearum* strain 23-10BR but shared no sequence homology to any other *Ralstonia* phages including RSA1.

#### (ii) Structure

Twenty-one ORFs were predicted to be involved in morphogenesis of phage RsoM1USA including 6 for capsid- and 15 for tail-related proteins (Supplementary Table S1). ORFs encoding phage capsid-related proteins included ORF 6 to ORF 11 for putative portal protein (ORF6), terminase (ORFs 7 and 10), scaffolding protein (ORF8), major capsid protein (ORF9), and head completion/stabilization protein (ORF11). ORFs encoding phage tail-related proteins included ORFs 12, 17, 18, 23, 29 to 32 for tail, ORFs 20 to 22 for baseplate, ORFs 24 and 25 for tail fiber, and ORFs 27 and 28 for tail sheath proteins. All structural genes of phage RsoM1USA shared aa sequence identity of 70 to 97% with the corresponding ORFs in phage RSA1 (Figure 2 and Supplementary Table S1). The genomic organization of the structural genes in the two phages is also very similar, except that ORF19, located between the structural genes *orf18* and *orf20* in phage RsoM1USA, was annotated as a hypothetical protein and did not share any aa sequence homology with the similarly located ORF18 in phage RSA1 (Figure 2 and Supplementary Table S1), which was also annotated as a hypothetical protein in phage RSA1 (Fujiwara et al., 2008).

**FIGURE 3:**
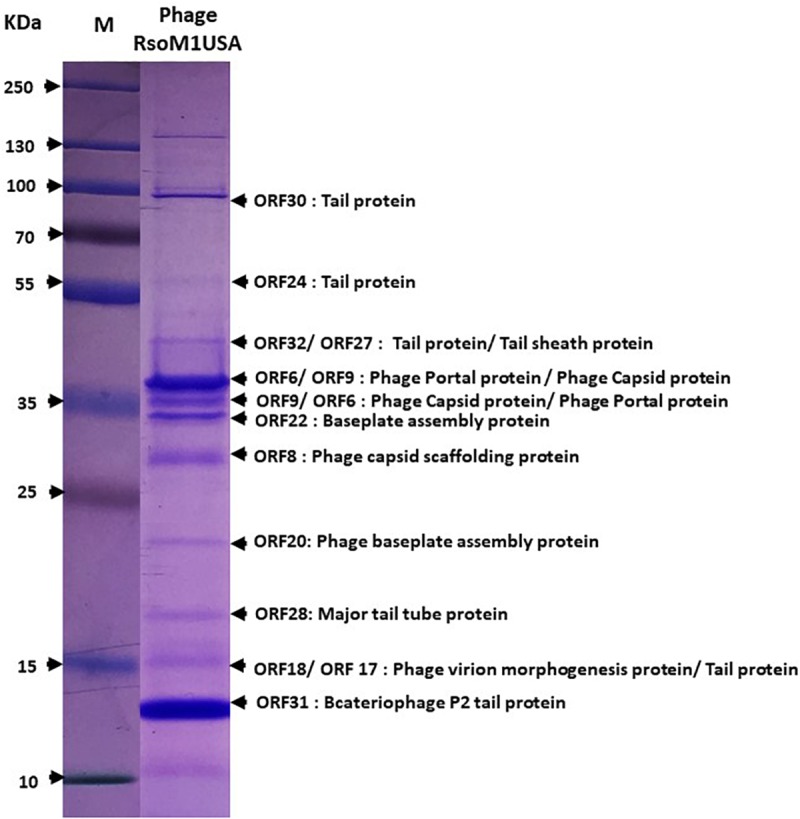
Expression of structural proteins of Ralstonia phage RsoM1USA. Proteins from purified phage virions were separated by SDS-PAGE gel (14%) and stained with Coomassie blue. The functions of the 11 protein bands predicted to correspond to ORFs 6, 8, 9, 17, 18, 20, 22, 24, 28, 30, 31, and 32 were indicated. M: protein ladder, with its molecular weight in kilodaltons (kDa) indicated on the left.

To confirm the identity of the major structural components of phage RsoM1USA, proteomic analysis of the purified phage virions was performed by SDS-PAGE gel. At least 11 proteins ranging from 15 to over 90 kDa were separated in the gel (Figure 3). These bands were cut out and sequenced commercially. Based on the peptides identified using mass spectrometry and their relative abundance, as well as comparison to the deduced amino acid sequences and molecular masses of ORFs in phage RsoM1USA, the 11 bands most likely correspond to phage tail (ORFs 17, 24, 30 and 32), tail sheath (ORF27), portal (ORF 6), capsid (ORF9), baseplate assembly (ORFs 20 and 22), capsid scaffolding (ORF8), major tail tube (ORF28), virion morphogenesis (ORF 18), and bacteriophage P2 tail (ORF 31) proteins (Figure 3).

#### (iii) Lysis and Integration

Four consecutive ORFs (ORFs 13 to 16) were predicted to be involved in lysis of bacterial cells. ORFs 13 and 14 were annotated to encode phage-related transmembrane proteins, sharing aa sequence identity of 100% to the membrane protein and to the phage holin family protein, respectively, of *R. solanacearum* strain 23-10BR (Supplementary Table S1). ORF15 was predicted to be a peptidoglycan-binding protein (99% identity to *R. solanacearum* strain 23-10BR sequence) (Supplementary Table S1). ORF16 shared high aa sequence identity to a peptide in *R. solanacearum* strain 10-23BR (Supplementary Table S1), that may be involved in host lysis through endolysins with signal peptides (Catalao et al., 2013).

ORF56 of phage RsoM1USA was annotated as an integrase, as was ORF50 in phage RSA1. Surprisingly, however, the two integrase sequences did not share any aa sequence identity (Figure 2).

#### (iv) Hypothetical and Other Proteins

Twenty-four ORFs of phage RsoM1USA were annotated as hypothetical proteins, or proteins with other functions. Nineteen ORFs had homologs in phage RSA1, but ORFs 3, 19, 38, 39, and 49 did not. ORF55 was located before and ORFs 57 and 58 after the integrase (ORF56) and *att*P region in phage RsoM1USA, while no counterpart for ORF55 was present and only one ORF (ORF51) was found after the integrase (ORF50) and *att*P region in phage RSA1 (Figure 2). In addition, ORFs 57 and 58 of phage RsoM1USA did not share any aa sequence homology with ORF51 of phage RSA1 (Figure 2).

### Phylogenetic Relationships Between Phages RsoM1USA and RSA1

**FIGURE 4:**
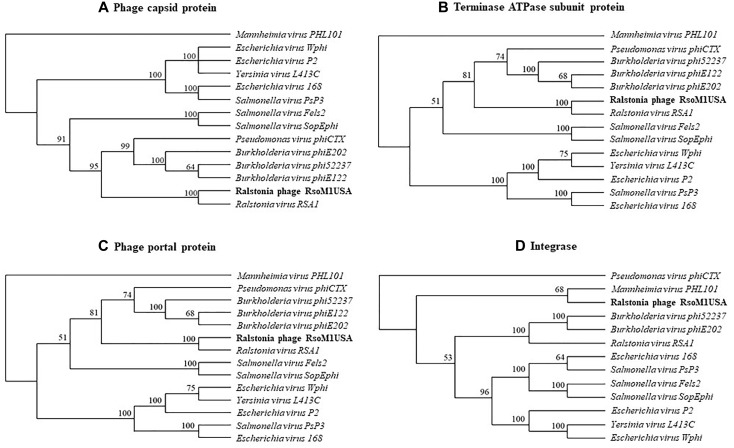
Phylogenetic relationships between Ralstonia phage RsoM1USA and 13 other phages of the genus *P2virus* (under the subfamily *Peduovirinae* of the family *Myoviridae*) including *Ralstonia virus RSA1* (phage RSA1), based on deduced amino acid sequences of phage capsid protein **(A)**, terminase ATPase subunit protein **(B)**, and phage portal protein **(C)**, as well as between RsoM1USA and 12 phages based on annotated integrase **(D)**. The phylogenetic trees were generated using the downloadable alpha-test version of PAUP (Phylogenetic Analysis Using PAUP) (http://phylosolutions.com/paup-test). Vertical distances are arbitrary, but the horizontal branches are proportional to genetic distance. Bootstrap values (1,000 replicates) are represented at the nodes of the branches.

Phylogenetic relationships were determined among phage RsoM1USA and 13 members of the genus *P2virus* under the subfamily *Peduovirinae* of the family *Myoviridae* (Figure 4). A phylogenetic tree generated based on the deduced aa sequences of phage capsid proteins showed that phage RsoM1USA is more closely related to Ralstonia phage RSA1 than to the other 12 members of the *P2virus* (Figure 4A). Similar trees were also obtained when the deduced aa sequences of terminase ATPase subunit and phage portal proteins were used, respectively (Figure 4B,C). Phage RsoM1USA was shown to be more closely related to *Mannheimia virus PHL101* than to phage RSA1, however, when the deduced aa sequences of the integrase protein was used for phylogenetic analysis (Figure 4D).

### Integration and Attachment Site for Phage RsoM1USA

**FIGURE 5:**
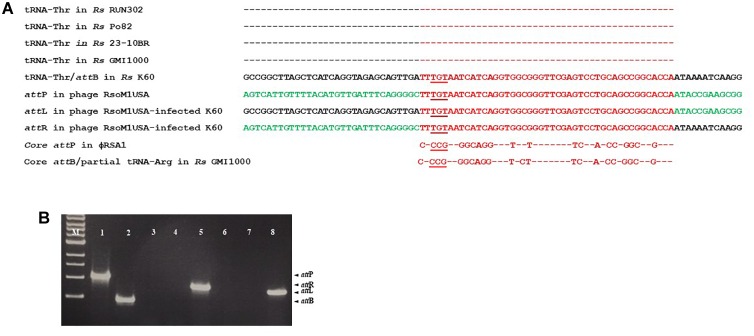
Integration of Ralstonia phage RsoM1USA into the genome of *R. solanacearum* strain K60. **(A)** Comparison of the attachment (*att*) sequences. The *att*B sequence of *R. solanacearum* strain K60 (before phage RsoM1USA integration) contains the same 76-bp threonine (Thr) tRNA sequence found in *R. solanacearum* strains RUN302, P082, 10-23BR, and GMI1000 (the same amino acid is indicated by “–”). The *att*B sequence in *R. solanacearum* strain K60 was aligned with the tRNA-threonine-like *att*P sequence in Ralstonia phage RsoM1USA. The 54-bp core *att* sequence is shown in red. The sequences after integration of phage RsoM1USA into *R. solanacearum* strain K60 (*att*L and *att*R) are shown. Sequences from the bacterial strain K60 and the phage RsoM1USA are shown in black and green, respectively. The core *att*P sequence of phage RsoM1USA was also compared to that of phage RSA1 which matched the 3′ arginine (Arg) tRNA in *R. solanacearum* strain GMI1000 (Fujiwara et al., 2008). Only nucleotides different from phage RsoM1USA’s core *att*P sequence were shown. The anticodons of threonine (TGT) and arginine (CCG) tRNAs are underlined. *Rs*: *R. solanacearum*. **(B)** Amplification of *att* regions by PCR and separation by gel electrophoresis. The 1,068-bp *att*P and 452-bp *att*B fragments were amplified from DNA of phage RsoM1USA (lane 1) and *R. solanacearum* strain K60 (lane 2), respectively. The 800-bp *att*R and 720-bp *att*L fragments were detected only from DNA of phage RsoM1USA-infected strain K60 (lanes 5 and 8) after integration, but not from phage RsoM1USA (lanes 3 and 6) and strain K60 (lanes 4 and 7). M: 1-kb DNA ladder.

By BLAST search, we identified a tRNA-threonine-like sequence, corresponding to nucleotides 36,657 to 36,732, in phage RsoM1USA (Figure 5A). This sequence matched 100% to the 45 nucleotides at the 3′ end of the 76-bp threonine tRNA sequences in *R. solanacearum* strains RUN302, Po82, 23-10BR, GMI1000 and K60 (Figure 5A), suggesting that phage RsoM1USA contains a *att*P site where it recombines with the homologous bacterial *att*B site for the phage to integrate into the bacterial genome. To confirm that this 45-bp core sequence is involved in the integration of phage RsoM1USA into its susceptible *R. solanacearum* strain K60, the left and right integration flanking regions, *att*L and *att*R, were amplified by PCR using DNA of phage RsoM1USA-infected K60 as a template (Figure 5B) followed by sequencing of the PCR products (Figure 5A). Both the *att*L and *att*R fragments contained the 45-bp core *att* sequence that is in both the bacterial K60 and the phage RsoM1USA genomes (Figure 5A). The *att*L fragment contained K60 genome sequence upstream and phage RsoM1USA sequence downstream of this core sequence (Figure 5A). The *att*R site had phage RsoM1USA sequence upstream and K60 sequence downstream of the core sequencing (Figure 5A), the reverse of the orientation found for the *att*L site, confirming that the 45-bp core sequence is the site of phage integration.

### Effects of Phage RsoM1USA on *R. solanacearum* Strain RUN302 *in vitro* and in planta

**FIGURE 6:**
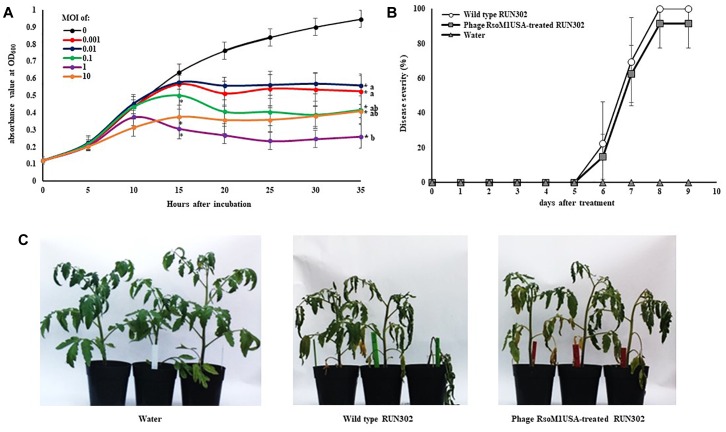
Effects of Ralstonia phage RsoM1USA on *R. solanacearum* strain RUN302. **(A)** *In vitro* growth of *R. solanacearum* strain RUN302 inoculated with phage RsoM1USA at a multiplicity of infection (MOI) of 0 (black), 0.001 (red), 0.01 (blue), 0.1 (green), 1 (pink) and 10 (orange), respectively. Bacterial growth indicated by absorbance at 600 nm was measured every 60 min and graphed every 5 h for 35 h. Points shown are the means of three separate experiments, each containing three replicates for each MOI treatment. Bars indicate standard errors. ^∗^ indicates significant difference (*p* < 0.05) at hours 15 and 35 between MOI of 0 and each of the other MOIs by the *t*-test. Means with different letters at hour 35 are significantly different based on the Tukey’s Honest Significant Difference test (*p* < 0.05). **(B)** Virulence of *R. solanacearum* strain RUN302 alone (open circle) and co-inoculated with phage RsoM1USA at the MOI of 0.1 (closed square). Disease severity was calculated based on a 0 (healthy) to 4 (75 to 100% leaves wilted) disease index of each plant. Points shown are means of three separate experiments, each containing 12 plants per treatment. Bars indicate standard errors. Significance of means was measured using the *t*-test (*p* < 0.05). **(C)** Appearance of the inoculated plants 8 days after soil drenching inoculation with water (left), RUN302 (middle), or RUN302 followed by phage RsoM1USA at the MOI of 0.1 (right).

To study the effect of phage RsoM1USA on the *in vitro* growth and virulence of its susceptible *R. solanacearum* strain RUN302, we compared the *in vitro* growth and virulence of the wild type RUN302 to those of the phage-treated RUN302 of *R. solanacearum*. Strain RUN302 was chosen for the study because it showed the highest efficiency of phage infection (Table 1) and did not contain any intact prophages like prophage RsoM1USA in its genome before phage treatment. The growth of phage-treated *R. solanacearum* strain RUN302 was significantly reduced 15 h after phage treatment at MOI of 0.1, 1, and 10 (*p* < 0.05), and 35 h at all tested MOIs (*p* < 0.05) (Figure 6A). Since MOIs of 10, 1 and 0.1 resulted in similarly reduced bacterial growth 35 h after the phage treatment (*p* < 0.05) (Figure 6A), the lowest MOI of 0.1 was chosen for virulence assays in tomato plants for practical purposes. Results from our virulence assays revealed that both the untreated and the phage-treated RUN302 strains started to wilt tomato plants 6 days after soil drenching inoculation, and reached disease severity of over 80% by day 8 (Figure 6B,C). The overall disease severity caused by untreated RUN302 was not statistically different from that caused by the phage-treated RUN302 (*p* = 0.423 by the *t*-test at day 9) (Figure 6B). Tomato plants inoculated with water displayed no disease symptoms (Figure 6B,C).

## Discussion

RsoM1USA is the first *Ralstonia*-infecting phage isolated from soil of the United States from a tomato field in Florida. We purified the phage and determined the phage to be a member of the genus *P2virus* in the family *Myoviridae* based on its morphology. The phage was designated RsoM1USA using the systematic phage identifier naming approach we proposed previously to make the phage name not only unique but also meaningful regarding the phage’s host species, morphology and origin (Ahmad et al., 2018).

Phage RsoM1USA contains all genes responsible for the morphogenesis of the phage head or capsid assembly as described by Fujiwara et al. (2008) and Fokine and Rossmann (2014), and those genes have high sequence homology (70–97%) with those of phage RSA1. It is, therefore, not surprising that virions of phage RsoM1USA resemble those of phage RSA1 (Yamada et al., 2007), displaying an icosahedral capsid and a long contractile tail with a tail sheath. Like phage RSA1, phage RsoM1USA encodes putative lysis genes commonly found in P2-like phages (Christie and Calendar, 2016) such as holin, suggesting that RsoM1USA may lyse host cells using holin and endolysin. Holin plays a key role in host lysis by forming large pores that are utilized as transport channels for endolysin to access and degrade the peptidoglycan layer resulting in host lysis (Shin et al., 2014). It is worth noting that both phages contain ORF1, encoding a protein similar to the PAAR (proline-alanine-alanine-arginine)-domain containing protein. This protein has been found mostly in beta- and gamma-proteobacteria such as *Serratia marcescens, Vibrio cholerae*, or *Acinetobacter baylyi* (Shneider et al., 2013; Cianfanelli et al., 2016). It is known to be a component of the spike complex of the bacterial type VI secretion system (T6SS) and has been reported to play a role in bacterial virulence (Shneider et al., 2013; Cianfanelli et al., 2016). In addition to the similarity in gene organization, the two phages are also similar in size (39,309 vs. 38,760-bp), and are closely related to each other (Figure 4A–C). Since phages RsoM1USA and RSA1 were isolated from soil in the United States and Japan, respectively, it raises an interesting question how the phages obtained from two different countries across continents share morphological and genomic similarities. It is likely that the two phages share common ancestry, as Hendrix et al. (1999) proposed for “all of the dsDNA tailed phages.” Many ORFs of phage RsoM1USA also have high sequence identity with prophage sequences in *R. solanacearum* strain 23-10BR (Supplementary Table S1), which is a sequevar 27 strain isolated from Brazil (Clarke et al., 2015), suggesting evolutionary relationships between the phage and bacterial strain.

Despite the similarities between phages RsoM1USA and RSA1, the two phages are different in the following ways: (1) The genes for metabolism are highly variable between the two phages. Among the eight ORFs annotated to have metabolic functions in phage RsoM1USA, one (ORF 35) shared low sequence homology with and four (ORFs 33, 36, 37, and 57) have no counterpart ORFs in phage RSA1 (Figure 2). Instead, they share some sequence homology with *R. solanacearum* strains including 23-10BR and CQPS-1 (Supplementary Table S1). (2) Seven ORFs in phage RsoM1USA annotated as hypothetical/other proteins have no corresponding ORFs in phage RSA1. (3) Myoviruses are known to integrate into bacterial genomes mediated by a portion of bacterial tRNA, an *att* site, as in the cases of phages RSA1 and RSY 1 of *R. solanacearum* (Fujiwara et al., 2008; Askora et al., 2017), phage 16-3 of *Rhizobium meliloti* (Blaha et al., 2004), and phage vB_RleM_PPF1 on *Rhizobium leguminosarum* (Halmillawewa et al., 2016). We confirmed experimentally that phage RsoM1USA uses the 3′ 45-base portion of the threonine tRNA (TGT) gene as its *att* site (Figure 5), which is different from RSA1 which uses the 3′ 45-base of the arginine tRNA (CCG) gene (Fujiwara et al., 2008). This is also different from the *Ralstonia*-infecting myovirus RSY1 which uses a 3′ 15-base portion of the serine tRNA (GGA) gene for integration and contains an integrase gene in opposite orientation (Askora et al., 2017). In addition, putative integrases encoded by ORF56 of phage RsoM1USA and ORF 50 of phage RSA1 have no sequence identity. The phylogenetic trees derived from the deduced amino acid sequences of capsid, terminase ATPase subunit and portal proteins all suggest that phage RsoM1USA is more closely related to the Ralstonia phage RSA1 than to other myoviruses in the genus *P2virus* (Figure 4A–C). A phylogenetic tree derived from deduced amino acid sequences of integrase, however, revealed that RsoM1USA is more closely related to *Mannheimia virus PHL101* than to phage RSA1 (Figure 4D). These results suggest that integrases in the two phages may be evolved from different sources and they use different mechanism for integration. In view of these differences, phage RsoM1USA is either a variant of phage RSA1 or represents a third group, the RsoM1USA-type, in the p2-like lineages of *R. solanacearum* phages. At the species level, phages RsoM1USA and RSA1 are different based on their 89% nucleotide sequence identity, which is less than the 95% identity in DNA sequence conventionally used as a criterion to be considered the same species (Addy et al., 2018). As a result, the Ralstonia phage RsoM1USA should be considered a member of a new species with a proposed species name of *Ralstonia virus RsoM1USA* under the genus *P2virus* in the family *Myoviridae*.

Phage RsoM1USA is capable of infecting strains belonging to different *Ralstonia* species and originated from different geographic locations. Out of the 30 tested *R. solanacearum* species complex strains collected worldwide, 12 were susceptible to phage RsoM1USA including biovars 1, 2, 2T and 3 strains in two of the recently classified *Ralstonia* species: the current *R. solanacearum* (phylotypes II and III) and *R. pseudosolanacearum* (phylotypes I) originally isolated from the United States, Brazil, United Kingdom, Netherlands, Guatemala, and Taiwan (Table 1). It is worth noting that previous host range studies for *Ralstonia* phages used strains isolated only from the testing country (Yamada et al., 2007; Murugaiyan et al., 2010). In addition, those tested bacterial strains belong only to the current *R. pseudosolanacearum* species, making it harder to assess the biocontrol potential of the *Ralstonia* phages on *Ralstonia* strains beyond that country and species.

Compared to the untreated *R. solanacearum* strain RUN302, the *in vitro* growth of phage RsoM1USA-treated *R. solanacearum* strain RUN302 was significantly reduced when the phage was added to the bacterial cells at MOIs of 0.001 to 10. We used MOI of 0.1, not a higher one like 1 or 10, in our virulence assay is because by the Tukey’s Host Significant Difference test for the five MOIs used (0.001, 0.01, 0.1, 1, and 10), no significant difference in *in vitro* growth of *R. solanacearum* strain RUN302 treated by phage RsoM1USA was found at a MIO of 0.1, 1 or 10. We therefore chose the lowest MOI for practical purposes. The disease severity caused by the phage-treated RUN302 strain, however, was not affected in tomato plants. This suggests that the activity of a phage *in vitro* on a synthetic medium is not always correlated with its activity *in planta* under artificial or natural environment (soil or potting mix). The latter is chemically and physically more complex and may affect phage-bacterial host interaction and phage infectivity, particularly during phage absorption stage (Marsh and Wellington, 1994). Kimura et al. (2008) found that phage particles can be absorbed to soil with the degree of absorption over 90%, therefore decreasing the number of free phage particles available to infect bacterial hosts.

By BLAST search, the 45-bp *att*P site was found with 100% identity to the 45 nucleotides at the 3′ end of the 76-bp threonine tRNA sequences corresponding to nucleotide 1,494,395 to 1,494,470 in *R. solanacearum* strain RUN302 [(with an alternative name of IBSBF 1503 (Wicker et al., 2007)] (Figure 5), suggesting that phage RsoM1USA is capable of integrating into the genome of RUN302, causing no significant effect on bacterial virulence. This is similar to what was found for Ralstonia phage RSA1, the most closely related phage to RsoM1USA. Phage RSA1 uses the 3′ 45-base arginine gene for integration and results in no significant changes in virulence with RSA1 lysogenic cells *in planta* (Fujiwara et al., 2008). This, however, would be different from previously characterized lysogenic bacteriophages, which have been demonstrated under greenhouse conditions to either significantly enhance virulence (e.g., Ralstonia phage RSS1 in Addy et al., 2012b) or reduce virulence (e.g., Ralstonia phage Rs551 in Ahmad et al., 2017b and Xanthomonas phage XacF1 in Ahmad et al., 2014). Ralstonia phage RSM3 even caused loss of virulence of phage-infected *R. solanacearum* strain MAFF106611 (Addy et al., 2012a), but further studies are needed to develop this phage into a biocontrol agent. Whether these are the reasons why phage RsoM1USA did not prevent plant disease caused by *R. solanacearum* is worthy of future research. Future research is also needed to determine whether phage RsoM1USA plays an important ecological role in “regulating the virulence of and offering competitive fitness to its carrier bacterial strain for persistence of the bacterium in the environment,” similar to what Ralstonia phage Rs551 does (Ahmad et al., 2017a). These studies are important for a better understanding of the relationships among phages, bacterial hosts and their environments for effective control of *R. solanacearum*.

## Conclusion

A *Ralstonia*-infecting bacteriophage designated RsoM1USA was isolated from soil of the United States. It is a member of *Myoviridae* containing 39,309 nucleotides with 58 ORFs. It is closely related to Ralstonia phage RSA1 but is different from RSA1 mainly in integrase and in utilizing the threonine tRNA as the *att* region for integration. Phage RsoM1USA should be considered a member of a new species with a proposed species name of *Ralstonia virus RsoM1USA.* Although phage RsoM1USA significantly reduced the *in vitro* growth of *R. solanacearum* strain RUN302 at MOIs between 0.001 and 10, it had no significant effect in reducing disease symptoms of RUN302 in tomato plants at MOI of 0.1 when compared to the untreated controls. The role of phage RsoM1USA, if any, in providing competitive fitness to its carrier bacterial strains remains to be determined.

## Author Contributions

HA, AA, and QH conceived and designed the experiments and analyzed the data. HA and AA performed the experiments. QH contributed to the reagents, materials, and analysis tools. HA and QH wrote the manuscript.

## Conflict of Interest Statement

The authors declare that the research was conducted in the absence of any commercial or financial relationships that could be construed as a potential conflict of interest.

## Footnotes

1 http://lowelab.ucsc.edu/tRNAscan-SE/

2 https://www.ebi.ac.uk/Tools/msa/clustalo

3 http://phylosolutions.com/paup-test

4 http://vassarstats.net/anova1u.html
