# Supplementary material for: Molecular and Biological Characterization of Ralstonia Phage RsoM1USA, a New Species of P2virus, Isolated in the United States
Source: Front Microbiol. 2019 Feb 19;10:267. doi: 10.3389/fmicb.2019.00267 (PMC6389784; doi:10.3389/fmicb.2019.00267)
Supplement: Supplementary file 1 [file Table_1.docx]

**Table S1. List of ORFs of phage RsoM1USA and their BLASTp results.**

| **Coding sequence** | **Strand** | **start** | **stop** | **Length (aa)** | **Mass (Da)** | **Predicted functional role** | **E-value** | **Accession No.** |
| --- | --- | --- | --- | --- | --- | --- | --- | --- |
| ORF1 | + | 350 | 742 | 130 | 13,231 | PAAR domain-containing protein /*R. solanacearum* strain 23-10BR | 2.00E-87 | WP_080727940.1 |
| ORF2 | + | 744 | 1,247 | 167 | 18,741 | DUF4123 domain-containing protein/ *R. solanacearum* strain 23-10BR | 7.00E-115 | WP_039572636.1 |
| ORF3 | + | 1,234 | 1,581 | 115 | 13,001 | hypothetical protein/ *R. solanacearum* strain 23-10BR | 9.00E-78 | WP_039572632.1 |
| ORF4 | + | 1,581 | 2,189 | 202 | 22,230 | hypothetical protein/*R. solanacearum* strain 23-10BR | 8.00E-147 | WP_039565397.1 |
| ORF5 | + | 2,099 | 2,914 | 271 | 30,140 | hypothetical protein/ *R. solanacearum* strain 23-10BR | 0.00E+00 | WP_039565399 |
| ORF6 | - | 2,899 | 4,005 | 368 | 41,528 | phage portal protein/ *R. solanacearum* strain FJAT-91 | 0.00E+00 | WP_086706305.1 |
| ORF7 | - | 4,002 | 5,783 | 593 | 66,942 | oxidoreductase, Terminase ATPase subunit/*R. solanacearum* strain 23-10BR | 0.00E+00 | WP_039572630 |
| ORF8 | + | 5,927 | 6,772 | 281 | 30,389 | phage capsid scaffolding protein *R. solanacearum* strain 23-10BR | 0.00E+00 | WP_039572627 |
| ORF9 | + | 6,826 | 7,842 | 338 | 37,795 | phage capsid protein/ *R. solanacearum* strain 23-10BR | 0.00E+00 | WP_039572624 |
| ORF10 |  | 7,839 | 8,561 | 240 | 26,608 | terminase endonuclease subunit/ *R. solanacearum* strain 23-10BR | 1.40E-168 | WP_039572620.1 |
| ORF11 | + | 8,657 | 9,136 | 159 | 17,134 | head completion/stabilization protein/ *R. solanacearum* strain 23-10BR | 2.00E-109 | WP_039572616.1 |
| ORF12 | + | 9,136 | 9,342 | 68 | 7,278 | tail protein X/ *R. solanacearum* strain 23-10BR | 2.00E-41 | WP_039572614 |
| ORF13 | + | 9,358 | 9,753 | 131 | 12,755 | membrane protein/ *R. solanacearum* strain 23-10BR | 2.00E-80 | WP_039572612.1 |
| ORF14 | + | 9,750 | 10,064 | 104 | 11,133 | phage holin family protein/ *R. solanacearum* strain 23-10BR | 1.40E-64 | WP_039565349.1 |
| ORF15 | + | 10,061 | 10,867 | 268 | 28,638 | peptidoglycan-binding protein/ *R. solanacearum* strain 23-10BR | 0.00E+00 | WP_039572610 |
| ORF16 | + | 10,864 | 11,364 | 166 | 17,219 | signal peptide protein/ *R. solanacearum* strain 23-10BR | 2.00E-114 | WP_039572608 |
| ORF17 | + | 11,361 | 11,795 | 144 | 16,041 | tail protein/ *R. solanacearum* strain 23-10BR | 5.10E-102 | WP_039565819.1 |
| ORF18 | + | 11,792 | 12,238 | 148 | 16,559 | phage virion morphogenesis protein/ *R. solanacearum* strain 23-10BR | 7.00E-100 | WP_039572606 |
| ORF19 | - | 12,300 | 12,722 | 140 | 15,999 | hypothetical protein/ *R. solanacearum* strain 23-10BR | 2.00E-100 | WP_080727597 |
| ORF20 | + | 12,994 | 13,611 | 205 | 21,591 | phage baseplate assembly protein V/ *R. solanacearum* strain 23-10BR | 2.00E-147 | WP_039565574 |
| ORF21 | + | 13,608 | 13,955 | 115 | 12,544 | baseplate assembly protein/ *R. solanacearum* strain 23-10BR | 6.30E-75 | WP_039565576.1 |
| ORF22 | + | 13,958 | 14,866 | 302 | 32,406 | baseplate assembly protein/ *R. solanacearum* strain 23-10BR | 0.00E+00 | WP_039572604 |
| ORF23 | + | 14,859 | 15,476 | 205 | 22,331 | phage tail protein I/ *R. solanacearum* strain 23-10BR | 8.30E-146 | WP_039572602.1 |
| ORF24 | + | 15,483 | 17,057 | 524 | 54,986 | phage tail protein/ *R. solanacearum* strain 23-10BR | 0.00E+00 | WP_064297624 |
| ORF25 | + | 17,067 | 17,819 | 250 | 26,075 | tail assembly protein/ *R. solanacearum* strain 23-10BR | 3.00E-175 | KFX27177 |
| ORF26 | + | 17,816 | 18,280 | 154 | 16,704 | hypothetical protein/ *R. solanacearum* strain 23-10BR | 5.00E-108 | WP_052022788 |
| ORF27 | + | 18,379 | 19,554 | 391 | 42,382 | tail sheath protein/ *R. solanacearum* strain 23-10BR | 0.00E+00 | WP_039572594 |
| ORF28 | + | 19,586 | 20,095 | 169 | 18,800 | major tail tube protein/ R. *solanacearum* strain 23-10BR | 8.00E-121 | WP_039572592 |
| ORF29 | + | 20,171 | 20,497 | 108 | 11,691 | phage tail assembly protein/ *R. solanacearum* strain 23-10BR | 2.00E-69 | WP_039572590 |
| ORF30 | + | 20,592 | 23,258 | 888 | 93,725 | tail protein/ *R. solanacearum* strain CIP120 | 0.00E+00 | OAI58293 |
| ORF31 | + | 23,261 | 23,683 | 140 | 15,604 | bacteriophage P2 tail protein GPU/ *R. solanacearum* K60-1 | 3.00E-99 | CCF95826 |
| ORF32 | + | 23,680 | 24,807 | 375 | 40,626 | tail protein/ *R. solanacearum* strain 23-10BR | 0.00E+00 | WP_039565387 |
| ORF33 | + | 25,078 | 25,860 | 260 | 28,596 | site-specific DNA-methyltransferase/ *R. solanacearum* strain 23-10BR | 0.00E+00 | WP_039565643 |
| ORF34 | - | 25,776 | 26,531 | 251 | 26,641 | hypothetical protein/ *R. solanacearum* strain 23-10BR | 0.00E+00 | WP_039571744.1 |
| ORF35 | - | 26,690 | 27,127 | 145 | 15,437 | XRE family transcriptional regulator/ *R. solanacearum* strain 23-10BR | 3.00E-101 | WP_052491481.1 |
| ORF36 | - | 27,165 | 28,001 | 278 | 31,640 | IS3 family transposase/ *R. solanacearum* CQPS-1 | 0.00E+00 | WP_089191056 |
| ORF37 | - | 27,998 | 28,399 | 133 | 14,736 | IS2 insertion element repressor InsA; KpLE2 phage-like element/ *R. solanacearum* Phyl III-seqv23 | 2.00E-88 | CUV46884 |
| ORF38 | + | 28,527 | 28,730 | 204 | 7,522 | hypothetical protein/ *R. solanacearum* strain 23-10BR | 8.00E-39 | WP_080727568 |
| ORF39 | + | 28,757 | 28,948 | 63 | 7,221 | hypothetical protein/ *R. solanacearum* strain 23-10BR | 3.00E-37 | WP_039565595 |
| ORF40 | + | 28,977 | 29,171 | 64 | 7,423 | hypothetical protein *R. solanacearum* strain 23-10BR | 4.00E-36 | WP_039565590 |
| ORF41 | + | 29,174 | 29,416 | 82 | 9,222 | transcriptional regulator/ *R. solanacearum* strain 23-10BR | 1.00E-52 | WP_039565588 |
| ORF42 | + | 29,519 | 29,752 | 77 | 8,181 | hypothetical protein / *R. solanacearum* strain 23-10BR | 2.00E-47 | WP_039571724 |
| ORF43 | + | 29,749 | 29,913 | 54 | 5,642 | conserved protein of unknown function/ *R. solanacearum* Phyl III-seqv23 | 1.00E-22 | CUV28908 |
| ORF44 | + | 29,910 | 30,113 | 67 | 7,011 | hypothetical protein/ *R. solanacearum* strain 23-10BR | 1.00E-38 | WP_039571720 |
| ORF45 | + | 30,113 | 30,355 | 80 | 8,881 | hypothetical protein/ *R. solanacearum* strain 23-10BR | 1.00E-50 | WP_039571717 |
| ORF46 | + | 30,348 | 30,554 | 68 | 7,583 | hypothetical protein/ *R. solanacearum* strain 23-10BR | 1.00E-40 | WP_039571714.1 |
| ORF47 | + | 30,574 | 30,885 | 103 | 11,885 | hypothetical protein/ *R. solanacearum* strain 23-10BR | 1.00E-70 | WP_039565695 |
| ORF48 | + | 30,887 | 31,108 | 73 | 8,360 | hypothetical protein/ *R. solanacearum* strain 23-10BR | 5.00E-45 | WP_039565688 |
| ORF49 | + | 31,105 | 31,377 | 90 | 10,416 | hypothetical protein KR96_16665/ *R. solanacearum* strain 23-10BR | 3.00E-58 | KFX27856 |
| ORF50 | + | 31,374 | 31,664 | 96 | 11,105 | RNA-binding protein/ *R. solanacearum* strain 23-10BR | 4.00E-65 | KFX27855 |
| ORF51 | + | 31,664 | 34,447 | 927 | 103,074 | hypothetical protein/ *R. solanacearum* strain 23-10BR; DNA primase domain-containing protein | 0.00E+00 | WP_039571708 |
| ORF52 | - | 32,231 | 32,989 | 252 | 27,015 | hypothetical protein RPRSA1_gp48/ *R.* phage RSA1 | 1.00E-97 | YP_001165297 |
| ORF53 | - | 32,990 | 33,430 | 146 | 16,140 | hypothetical protein RPRSA1_gp48/ *R.* phage RSA1 | 5.00E-44 | YP_001165297 |
| ORF54 | + | 34,434 | 35,330 | 298 | 32,172 | hypothetical protein/ *R. solanacearum* FJAT-1458 | 3.00E-84 | WP_038961957 |
| ORF55 | + | 35,330 | 35,554 | 74 | 8,299 | DUF4224 domain-containing protein/ *R. solanacearum* strain 23-10BR | 2.00E-46 | WP_052022762 |
| ORF56 | + | 35,554 | 36,582 | 342 | 38,409 | integrase/ *R. solanacearum* strain 23-10BR | 0.00E+00 | KFX27852.1 |
| ORF57 | + | 37,103 | 38,149 | 348 | 38,113 | ParA family protein/ *R. solanacearum* strain 23-10BR | 0.00E+00 | WP_039572642 |
| ORF58 | + | 38,192 | 38,962 | 256 | 29,746 | DUF3800 domain-containing protein/ *R. solanacearum* strain 23-10BR | 0.00E+00 | WP_039572639 |
